# Ultrasound-Guided Diagnosis of Multiple Uterine Fibroids in a Patient With Polycystic Ovaries and Treatment With Relugolix, Estradiol, and Norethisterone Acetate

**DOI:** 10.7759/cureus.39055

**Published:** 2023-05-15

**Authors:** Joseph Acchiardo, Essence Maita, Ketan Jobanputra

**Affiliations:** 1 Radiology, Saint James School of Medicine, The Quarter, AIA; 2 Internal Medicine, Saint James School of Medicine, Arnos Vale, VCT; 3 Obstetrics and Gynecology, UChicago Medicine AdventHealth, Bolingbrook, USA

**Keywords:** estradiol / therapeutic use, leiomyoma / drug therapy, menorrhagia / drug therapy, ryeqo, myfembree, relugolix, norethindrone / adverse effects, uterine fibroid, polycystic ovary, ultrasound-guided

## Abstract

Uterine fibroids and ovarian cysts are common gynecological conditions that, while benign, when present simultaneously with bacterial vaginosis, can present a more complicated course of management. Symptoms of uterine fibroids include menorrhagia and dysmenorrhea, while ovarian cysts may present with pelvic pain and an adnexal mass. Each condition is typically managed separately; however, they can coexist in some patients, leading to a more complex presentation.

This case report presents a 35-year-old African American female patient with the simultaneous occurrence of uterine fibroids and ovarian cysts, complicated by recurrent vaginitis, along with the treatment approach. The treatment choice, relugolix, estradiol, and norethisterone (norethindrone) acetate, is the first once-daily U.S. Food and Drug Administration (FDA) combination hormonal medication approved for menorrhagia due to fibroids. This case is unique in that although the diagnoses are common, their coexistence makes for a more complex presentation, and the course of management presents a newly approved fixed-dose combination hormonal medication. This report discusses the incidence, pathophysiology, diagnosis, and management of uterine fibroids and ovarian cysts. Factors that may contribute to the concurrence of these conditions, such as genetic, hormonal, and environmental risks, are also explored. Diagnostic modalities and ultrasound techniques are reviewed, and treatment options, such as surgery and medical management, are discussed. The importance of a patient-centered approach in the treatment of multi-symptom gynecological disorders and the need to consider conservative management are emphasized.

## Introduction

Uterine fibroids are benign growths that originate from the smooth muscle cells of the uterus, while ovarian cysts are fluid-filled sacs that develop in the ovaries. Although both conditions are often non-cancerous, they can cause significant morbidity and may require intervention due to prolonged or heavy menstrual bleeding, pelvic pressure or pain, and, in rare cases, severe complications of pregnancy [1, 2]. Fibroids alone account for up to half of all hysterectomies and are associated with substantial healthcare costs for women of reproductive age [3].

The incidence of fibroid tumors among African American women by age 30 is 60%, increasing to over 80% by age 50, while Caucasian women have an incidence of 40% by age 35 and 70% by age 50 [4]. Worldwide, 20% of women are diagnosed with ovarian cysts before the age of 50 [2]. While the risks for developing polycystic ovaries are less clear, factors increasing fibroid risk include African American race, advanced age, premenopausal state, hypertension, family history, length of the postpartum period, certain food additives, and consumption of soybean milk. A decreased risk of fibroids was associated with oral contraceptive use, injectable contraceptive depot medroxyprogesterone acetate, and smoking in women with low body mass index and parity [5, 6].

Early diagnosis and management of uterine fibroids and ovarian cysts are essential for improving outcomes and reducing the risk of complications. Ultrasonography is the first-line imaging modality for examination and is considered the most rapid and convenient method of diagnosis for these conditions. Transvaginal ultrasound is regarded as a superior technique to abdominal ultrasound, with high sensitivity and specificity for diagnosis [7]. Treatment options may include medical management, surgery, or watchful waiting [8].

A new fixed-dose combination hormonal medication, which includes relugolix, estradiol, and norethisterone acetate, has recently received approval for treating uterine fibroid symptoms. This combination works by lowering ovarian hormone levels. In clinical trials, relugolix plus estradiol or norethisterone significantly reduced menstrual bleeding and improved other uterine fibroid-related symptoms in premenopausal women. The medication was well tolerated and did not cause significant bone loss, a known adverse effect of similar therapies. With their once-daily dosing regimen, relugolix, estradiol, and norethisterone acetate provide a viable treatment option for premenopausal women suffering from symptomatic uterine fibroids [9].

In this article, we will review the case of a premenopausal African American woman who was diagnosed with multiple uterine fibroids and polycystic ovaries via transvaginal ultrasonography. This case is unique in that this patient's treatment with a newly approved fixed-dose combination hormonal medication and the coexistence of bacterial vaginosis led to a more complex management course. This case highlights the importance of rapid ultrasound diagnosis and the consideration of uterine fibroids and ovarian cysts as potential causes of pelvic pain, menstrual abnormalities, and pregnancy complications, particularly in African American women who are at a higher risk for developing these conditions. Conservative and convenient treatment is often a priority for patients presenting with these symptoms.

## Case presentation

A 35-year-old African American female, gravida 4 para 1, presented to the OB-GYN clinic with complaints of pelvic pain, abnormal, painful uterine bleeding, and discharge for several weeks. Of her four previous pregnancies, three ended in miscarriage, with one full-term pregnancy yielding a viable fetus in 2015. The patient’s medical history was significant for an abnormal pap smear in 2013 and a loop electrosurgical excision procedure performed in 2014. The patient stated that since becoming pregnant in 2015, she has had recurrent bacterial vaginosis and discharge. She had a history of the human papillomavirus and the herpes simplex virus. She used condoms for contraception but had never been on oral contraceptive pills. Her monthly menstruation lasted six to seven days with menorrhagia and a varying frequency between 15 and 30 days. Her mother had a history of fibroids, and both her mother and maternal grandmother were diagnosed with type 2 diabetes mellitus.

**Figure 1:**
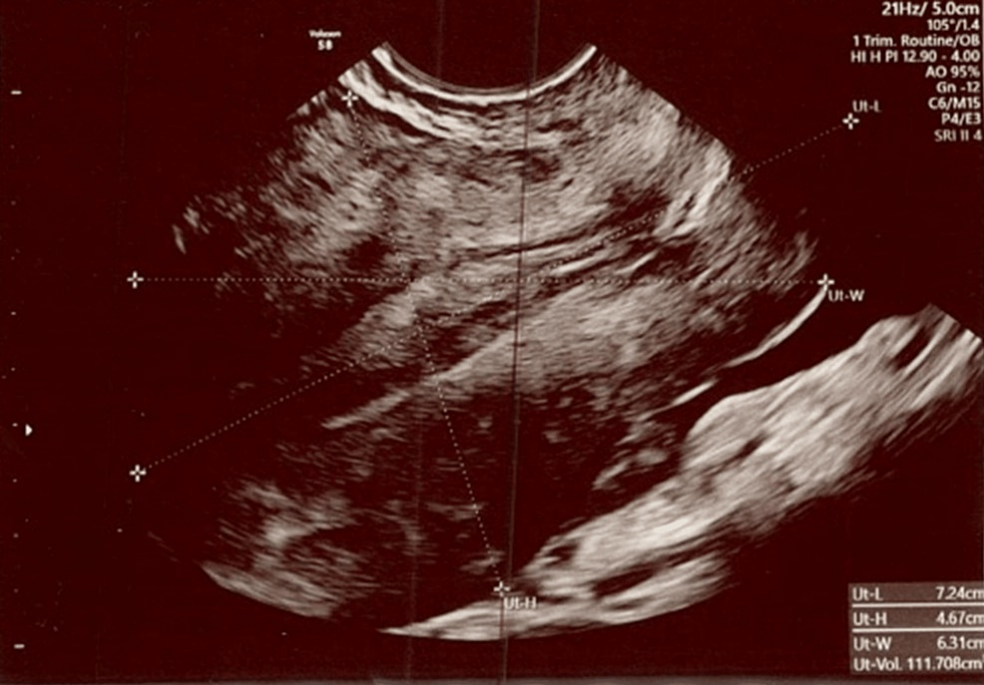
The uterus measures 7.2 cm x 4.7 cm x 6.3 cm, with a total volume of 111.7 cm3. Exam date: 04/17/2023; 2:22 PM Technique: transvaginal ultrasound

**Figure 2:**
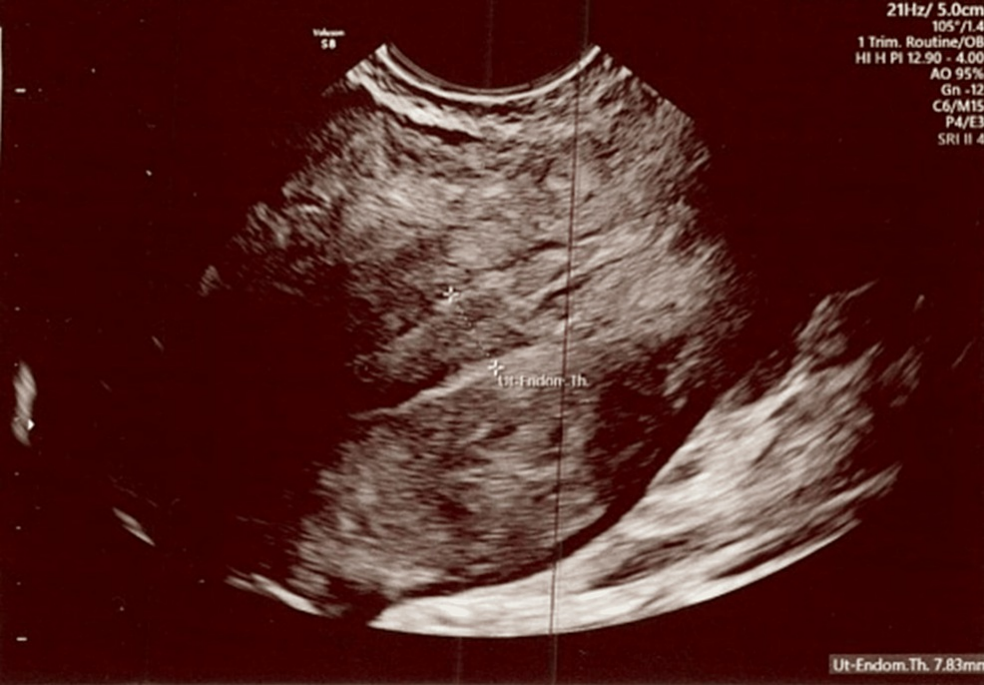
The ultrasound shows the total thickness of the uterus measured at 7.83 mm. Exam date: 04/17/2023; 2:22 PM Technique: transvaginal ultrasound

**Figure 3:**
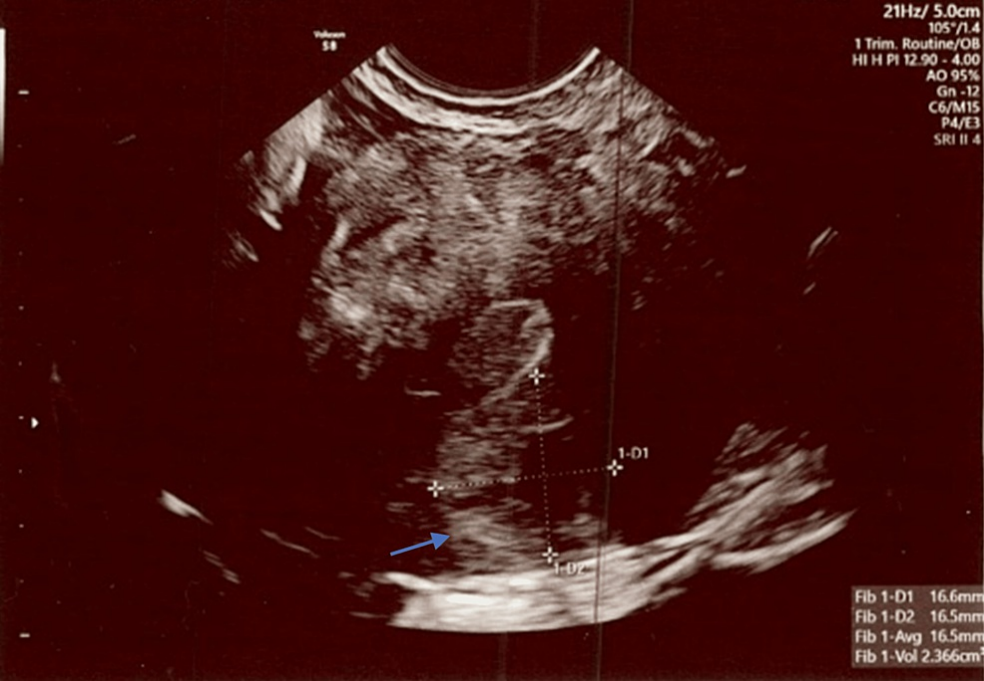
The ultrasound shows a posterior uterine fibroid measuring 16.6 mm x 16.5 mm with a 2.4 cm3 total volume. Exam date: 04/17/2023; 2:22 PM. Technique: transvaginal ultrasound

**Figure 4:**
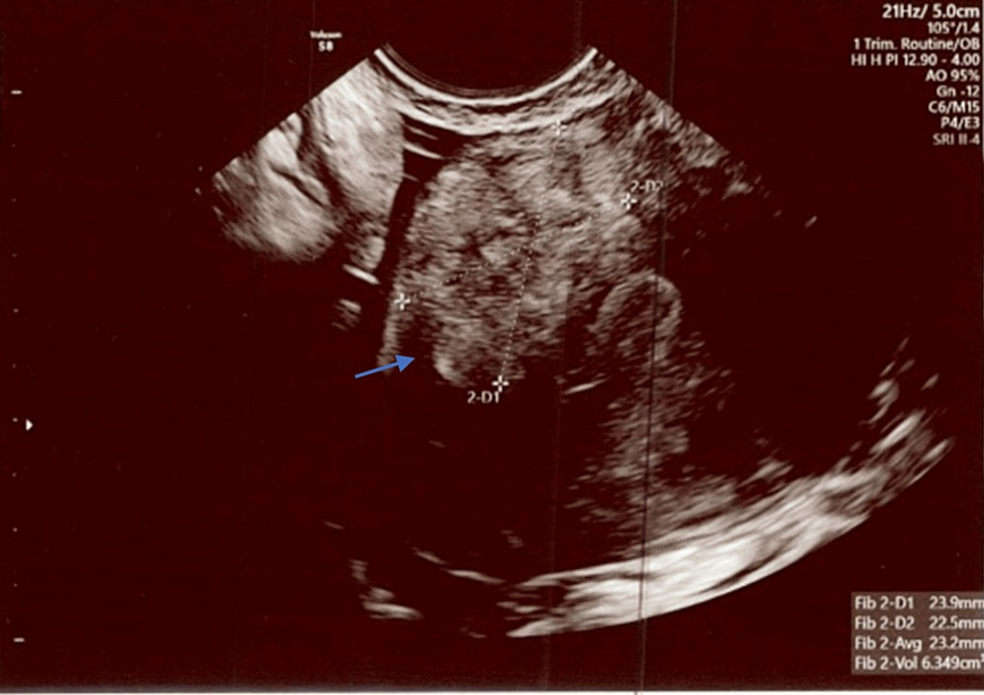
An anterior uterine fibroid measuring 23.9 mm x 22.5 mm with a 6.4 cm3 total volume was visualized. Exam date: 04/17/2023; 2:22 PM Technique: transvaginal ultrasound

**Figure 5:**
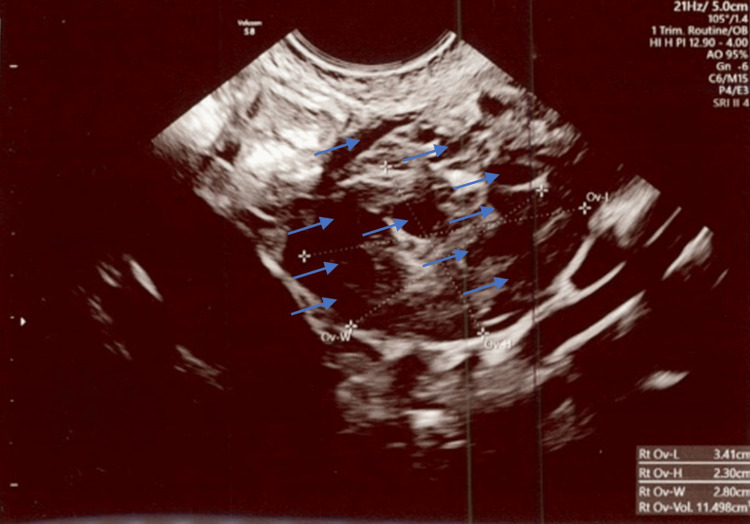
Polycystic right ovary measuring 3.4 cm x 2.3 cm x 2.8 cm and 11.5 cm3 in total volume with at least 10 visible follicles. Exam date: 04/17/2023; 2:22 PM. Technique: transvaginal ultrasound

**Figure 6:**
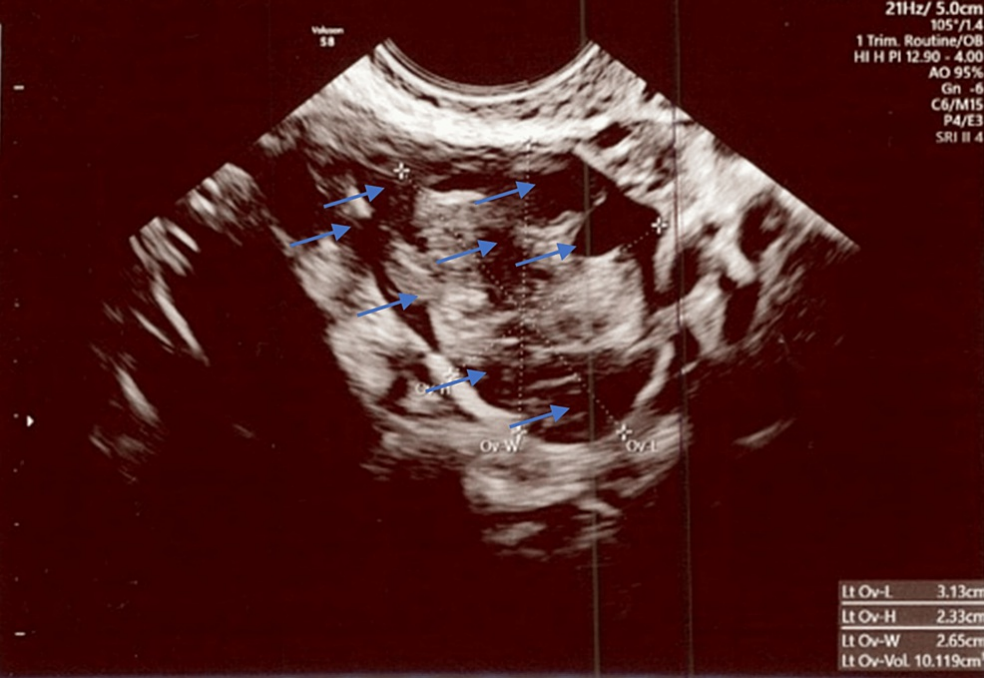
Polycystic left ovary measuring 3.4 cm x 2.3 cm x 2.8 cm and 11.5 cm3 in total volume with at least 8 visible follicles. Exam date: 04/17/2023; 2:22 PM Technique: transvaginal ultrasound

On examination, there was tenderness in the lower abdomen and blood in the vaginal cavity. The uterus was firm and immobile, with an irregular contour and several palpable anterior masses. A high vaginal swab (HVS) procedure was performed at the initial visit, and the patient was scheduled for a transvaginal ultrasound. At the following clinic visit, an ultrasound revealed the presence of three uterine fibroids and bilateral ovarian polycystic masses. Within the uterus, there were three fibroids: one posterior mass measuring 16 mm x 16 mm and two anterior masses measuring 23 mm x 22 mm and 16 mm x 18 mm. She was also found to have bilateral polycystic ovaries, with a right ovary volume of 11.5 mm3 and a left ovary volume of 10.1 mm3, as seen in Figures 1-6 below.

HVS returned positive for several bacteria, including *Atopobium vaginae*, Bacterial Vaginosis-Associated Bacterium 2 (BVAB2), *Mycoplasma hominis*, *Gardnerella vaginalis*, *Megaspheara type 1*, and U*reaplasma urealyticum*. This was treated with metronidazole 500mg twice a day for seven days, doxycycline hyclate 100mg twice a day for seven days, and fluconazole 200mg upon completion of other antibiotics.

The diagnosis, risks, and outcomes of uterine fibroids and polycystic ovaries and treatment options were thoroughly discussed with the patient, including medication management versus surgical intervention. Since the patient was under 45 years of age and interested in becoming pregnant again in the future, the decision was made to manage her symptoms conservatively with medication, routine follow-ups, and discussion of treatment options for fertility. Her symptoms were managed with the combination of hormonal medication relugolix, estradiol, and norethisterone acetate, one tablet daily for a trial of three months.

At her first two-week follow-up appointment, the patient reported a marked reduction in pelvic pain and discharge. At her one-month follow-up, the patient reported excitement for a four-pound weight loss and improvements in mood. Per the patient, there was a complete resolution of dysmenorrhea and menorrhagia. The patient also denied any vasomotor symptoms, nocturnal hyperhidrosis, or alopecia. At this visit, the patient was noted for having elevated blood pressure and was counseled on lifestyle modifications. Table 1 shows a record of the patient's vitals during clinic visits at various points in time. 

**Table 1:** Patient vitals during clinical visits BMI: body mass index; RR: respiratory rate; BP: blood pressure; HR: heart rate; SpO2: saturation of peripheral oxygen

Patient vitals								
	Weight _(lbs)_	BMI _(20-25)_	RR _(16-20 rpm)_	Systolic BP _(130-100 mmHg) _	Diastolic BP _(90-70 mmHg) _	HR _(60-100 bpm)_	Temperature _(97.6-99.6 F)_	SpO2 _(95-100%)_
_Day 1_	154.0	29.1	16	128	88	61	97.3	99
_Day 4_	154.0	29.1	16	126	84	74	97.4	98
_Day 14_	154.2	29.1	18	130	86	78	97.7	99
_Day 33_	150.6	28.3	16	136	90	66	97.2	99

She was scheduled for continued monitoring with her primary care physician.

## Discussion

Uterine fibroids occur with a higher incidence in African American women, and family history is a highly predictive risk factor, as seen in this patient [6]. These tumors cause a range of symptoms, including pelvic pain, heavy menstrual bleeding, and infertility [4]. In pregnancy, the presence of fibroids can lead to complications such as preterm labor, fetal malpresentation, and placental abruption [5]. Ovarian cysts, although common, have a less clear understanding of epidemiology, although their incidence seems to increase with age. Most ovarian cysts are functional and resolve spontaneously; however, some cysts can be complex and require further evaluation [2]. The presence of ovarian cysts in pregnancy can lead to complications such as torsion, rupture, and preterm labor [8]. Although most research has yielded inconclusive results on the relationship between the incidence of fibroids and polycystic ovaries, there is some evidence to suggest a connection between certain CA 125 and CA 19-9 positive ovarian tumors and uterine fibroids [10, 11]. One six-year longitudinal study involving 23,000 pre-menopausal African American women found that individuals with polycystic ovarian syndrome had a 65% greater incidence of fibroids compared to those without the condition. This phenomenon was attributed to the dysregulation of normal ovarian function due to elevated levels of androgens, ultimately leading to an increase in estrogen production, which in turn raises the likelihood of developing fibroids [12].

Given the high prevalence of common gynecological conditions, such as uterine fibroids and ovarian cysts, there is a need for highly accurate and rapid diagnostic modalities to facilitate their proper management. Ultrasonography is the primary imaging modality used for the diagnosis of these conditions, and it can be performed either through the transvaginal or abdominal route. Transvaginal ultrasonography is preferred for its higher sensitivity and specificity in detecting ovarian cysts and uterine fibroids, while abdominal ultrasonography is of limited value for assessing the size and location of these lesions in obese patients [7]. Other imaging modalities, such as MRI and CT scans, can be used in specific cases where ultrasound fails to provide clear and conclusive findings. A combination of imaging modalities and histopathological diagnostic measures can be utilized for the accurate diagnosis and management of uterine fibroids and ovarian cysts.

The treatment options for uterine fibroids and ovarian cysts depend on the size, location, and symptoms associated with the lesions. Conservative management with medications is usually the first-line approach for mild to moderate cases. The combination drug relugolix, estradiol, and norethindrone acetate was recently approved by the Food and Drug Administration (FDA) in May 2021 for the management of symptoms of dysmenorrhea and menorrhagia due to uterine fibroids in premenopausal women. Its mechanism of action involves inhibiting the production of gonadotropin-releasing hormone (GnRH), which reduces the production of estrogen and progesterone, the hormones that stimulate fibroid growth. It has been shown to significantly reduce menstrual bleeding while preserving bone mineral density in women with uterine fibroids [9]. Other medication treatment options include progestins, gonadotropin-releasing hormone agonists, and selective estrogen receptor modulators. However, these medications have limitations, including their side effect profile and the possibility of a return of symptoms after discontinuation.

Surgical treatment options are recommended for cases with severe symptoms or when medication therapy fails. Myomectomy, which involves the removal of the fibroid while preserving the uterus, is a preferred option for women who desire fertility. Hysterectomy, which involves the removal of the uterus, is recommended for women who have completed childbearing or have severe symptoms, such as leiomyoblastoma, and do not desire fertility [13, 14]. Salpingectomy, the removal of the fallopian tubes, is performed in cases of large or complex ovarian cysts, suspected malignancy, or risk reduction in women at elevated risk of ovarian cancer [15]. Interventional radiology offers minimally invasive treatment options for uterine fibroids. Uterine fibroid embolization (UFE) is an interventional radiology (IR) procedure that involves blocking the blood supply to the fibroids, causing them to shrink and die. UFE is effective in reducing fibroid size and improving symptoms while preserving the uterus, making it an attractive option for women who desire fertility [16].

## Conclusions

After careful patient-centered discussion and consideration, the decision was made to manage this patient conservatively with a three-month trial of the combination gonadotropin-releasing hormone (GnRH) inhibitors relugolix, estradiol, and norethisterone acetate for symptoms of menorrhagia and dysmenorrhea. Her bacterial vaginal infections were treated with a course of antibiotics and antifungals and a two-week follow-up. At the time the patient desires pregnancy, consideration of fertility treatment may be warranted and should be discussed within the context of a comprehensive evaluation of the patient's medical history of continuous vaginal infections and recurrent miscarriages.

Upon follow-up, the patient disclosed a complete resolution of symptoms and minimal side effects. She had no recurrence of vaginitis, discharge, or discomfort at a two-week or one-month follow-up. After one month, she reported weight loss, an improvement in her mood, and regular menstruation without dysmenorrhea or menorrhagia. She was noted to have elevated blood pressure on her return visit and was counseled on lifestyle modifications and scheduled with her primary care physician for further monitoring. At her three-month follow-up, the decision will be made to continue the patient on relugolix, estradiol, or norethisterone acetate or to discontinue and begin fertility treatment for the desired pregnancy.

## References

[1] Sohn GS, Cho S, Kim YM, Cho CH, Kim MR, Lee SR (2018). Current medical treatment of uterine fibroids. Obstet Gynecol Sci.

[2] Farghaly SA (2014). Current diagnosis and management of ovarian cysts. Clin Exp Obstet Gynecol.

[3] Stewart EA, Laughlin-Tommaso SK, Catherino WH, Lalitkumar S, Gupta D, Vollenhoven B (2016). Uterine fibroids. Nat Rev Dis Primers.

[4] Giuliani E, As-Sanie S, Marsh EE (2020). Epidemiology and management of uterine fibroids. Int J Gynaecol Obstet.

[5] Yang Q, Ciebiera M, Bariani MV, Ali M, Elkafas H, Boyer TG, Al-Hendy A (2022). Comprehensive review of uterine fibroids: developmental origin, pathogenesis, and treatment. Endocr Rev.

[6] Stewart EA, Cookson CL, Gandolfo RA, Schulze-Rath R (2017). Epidemiology of uterine fibroids: a systematic review. BJOG.

[7] Woźniak A, Woźniak S (2017). Ultrasonography of uterine leiomyomas. Prz Menopauzalny.

[8] Farkas AH, Abumusa H, Rossiter B (2023). Structural gynecological disease: fibroids, endometriosis, ovarian cysts. Med Clin North Am.

[9] Syed YY (2022). Relugolix/estradiol/norethisterone (norethindrone) acetate: a review in symptomatic uterine fibroids. Drugs.

[10] Mulita F, Liolis E, Kehagias D (2021). An enormous pelvic tumor in a 46-year-old woman with an elevated serum CA 125 level, what lies beneath it? Investigation of uterine tumors in postmenopausal women. Prz Menopauzalny.

[11] Mulita F, Oikonomou N, Tchabashvili L, Liolis E, Kehagias I (2020). A giant ovarian mucinous tumor in a 58-year-old postmenopausal patient with persistent abdominal pain and high serum levels of CA 19-9. Pan Afr Med J.

[12] Wise LA, Palmer JR, Stewart EA, Rosenberg L (2007). Polycystic ovary syndrome and risk of uterine leiomyomata. Fertil Steril.

[13] Henes M, Engler T, Taran FA, Brucker S, Rall K, Janz B, Lawrenz B (2018). Ovarian cyst removal influences ovarian reserve dependent on histology, size and type of operation. Womens Health (Lond).

[14] Mulita F, Iliopoulos F, Plachouri KM, Kehagias I (2021). Uterine leiomyoblastoma. BMJ Case Rep.

[15] Shi X, Chen S, Yang Y, Liu L, Huang L (2022). Laparoscopic surgeries for uterine fibroids and ovarian cysts reduce ovarian reserve via age- and surgical type-manner. Gynecol Endocrinol.

[16] Young M, Coffey W, Mikhail LN (2023). Uterine Fibroid Embolization. https://pubmed.ncbi.nlm.nih.gov/30085558/.

